# Associations

**Published:** 1860-07

**Authors:** 


### Associations.

### CINCINNATI DENTAL ASSOCIATION.

A regular meeting was held at the office of Dr. Richardson on the evening of June 12, 1860. *Present*—Drs. Taft, Richardson, Davenport, Wardle, Wells, and H. A. Smith.

Minutes of former meeting were read and approved.

Dr. H. A. Smith read an essay on the subject of filling teeth. This being the question for debate,

Dr. Taft said, in answer to the question to what extent the borders of the cavity should be cut away when they were thin and nothing remained but the enamel, that this depended somewhat upon the relative strength of the enamel substance. In some cases, the enamel was soft and friable and easily broken away, while in others, even a thin plate of enamel would resist a considerable amount of force, and he had frequently, in order to strengthen the walls of the cavity, where he feared they would be broken in consolidating the plug, moulded gutta percha over the tooth up to the edges of the cavity. In this way he filled cavities with thin walls with a feeling of security. The amount of pressure, he remarked, necessary to consolidate the plug, depended upon the size of the points of the pluggers used, and also upon the size of the pieces introduced for the filling.

Dr. Smith remarked that he was in the habit of cutting the walls of the cavity down, if practicable, until they presented a strong edge. The manner of filling the cavity would modify this to some extent, however. If blocks were used, more of the walls of the cavity would be required standing to retain the filling in place, than if adhesive foil was used. In block filling, the retention of the plug depended upon the several portions of gold introduced being kept bound together by the opposing walls of the cavity. But if adhesive foil be used, the plug being one solid mass, will be retained in a cavity comparatively superficial, if care is taken to secure well the first portions introduced. The borders of the cavity are much more liable to give way if blocks were used, but he would use this method in cases only where there was enough strength of wall to allow of sufficient pressure to insure a hard plug.

Dr. RICHARDSON described his method of filling crown cavities. If the cavity was of a considerable depth, he first introduced blocks of a length sufficient to fill one-half of the cavity. He first forms two opposing retaining points, in which he forces his first blocks, after which he introduces others in the usual way—consolidating them thoroughly—before he begins to fill the remainder of the cavity, which he does with adhesive foil. Does not think a filling made in this way as solid as if adhesive gold was used alone. He was led to adopt this method because he did not always succeed when starting a cavity with adhesive foil, in securing the first portions, so with retaining pits, that the entire mass of gold would not move in the cavity before the operation was finished. A tooth could be filled in this way in much less time than by the other method, and could be made sufficiently solid for all practical purposes. In filling a cavity upon the approximal surface of a bicuspid tooth, where the decay extended high up and under the gum, he introduced, after first carefully preparing the upper border of the cavity with well defined and square shoulders, a block of a size as large as the space would admit of, pressing it firmly in place. This afforded a basis for his filling, which he finished with adhesive foil, as before described.

Dr. SMITH spoke of the importance of adopting some one method of filling. It was in this way only that we could ever excel in this operation. Good plugs, it was true, could be made in the various methods in use. But he had observed that those who make uniformly good operations were those persons who had adopted a single method, and by long practice in it had become expert. He remembered having heard an operator of considerable reputation say that his success was due almost entirely to the fact of his having adopted the block method of filling, and that by long practice, he could now do what others fail in, who began the use of them at the same time, but abandoned them for other ways of filling.

Dr. TAFT described the method of securing a plug, if it moved slightly in the cavity before the gold is all introduced; it was by taking a fine wedge-shaped instrument, and passing several times around the edges of the filling, with considerable pressure. In this way it can be securely fixed in place. It was well, he remarked, after the cavity is full, to pass around the edges of the filling in the same manner, as it would produce a nice adaptation to the borders of the cavity, not attained in the ordinary way.

He concurred in what Dr. S. had said in relation to adopting a specific method of filling. For five years he used blocks in filling exclusively, and was confident it was not so good a method as the one he practiced now. He then mentioned some of the objections to block fillings. After abandoning blocks, he used crystal gold for three years, and since that time has used adhesive foil altogether. He was satisfied it was the best of all the methods he had tried.

Upon inquiry if any present were using os-artificial in fillings, Dr. TAFT mentioned that near six months ago he filled four molar teeth, which were much decayed, and portions of the walls broken away, so that the material was built up without much protection. He had seen these teeth within a few days past, and they seemed as perfect as at first. The plugs were exposed upon the masticating surfaces, and the teeth used freely.

Dr. SMITH had used it in a number of cases where the nerve was exposed a little, and in several instances where the tooth was aching slightly, with apparently good success. The feeling upon its first introduction, as described by patients, he thought similar to the action of chloride of zinc. He had made no examination to ascertain if the vitality of the teeth was destroyed.

Dr. DAVENPORT filled a tooth in his own mouth with this material some months ago. Occasionally there is a recurrence of the acrid taste experienced when the filling was first introduced. The surface at times he observes becomes roughened, owing perhaps, he said, to the peculiar condition of the secretions of the mouth.

Reports of cases being next in order. Dr. TAFT mentioned that he had recently filled two bicuspid teeth for a lady, and in excavating one of them there was one point in the cavity which was somewhat sensitive. The pulp was not exposed, and supposed it only sensitive dentine, it was not more sensitive than many that are successfully filled. Within a week after it was filled neuralgia set in upon that side of the face, the tooth seemed to be the starting point of the difficulty, though there was no soreness of the teeth. After a few days of ineffectual treatment removed the filling, and cut into the pulp and found it living, and very irritable; destroyed it and made a few applications of creosote into the fang, and after a few days filled. The patient has had no pain nor unpleasant symptoms since the tooth was filled.

Cases of the kind prompt us to more thorough examination before filling.

Dr. RICHARDSON reported a case where he had filled a tooth three years ago, and since then the patient had occasionally complained of pain in the tooth; but he did not think it sufficient to remove the plug, until recently when there was an increase of discomfort. When upon removing the plug and cutting into the pulp cavity, he found an entire absence of the remains of the pulp. How was the pulp removed and what was the cause of the pain.

The discussions being closed, Drs. H. R. SMITH and CAMERON were appointed essayists for the next meeting.

Filling teeth was continued as the subject for discussion at the next meeting.

The following persons was appointed delegates to the National Society to meet at Washington on Tuesday July 31, viz: Drs. H. R. SMITH, J. TAFT and H. A. SMITH.

The Society adjourned to meet at the office of Drs. WARDLE and DOUGHTY, on the second Tuesday, of July, at 8 o'clock P. M. H. A. SMITH, *Sec'y.*

### APPOINTMENT OF DELEGATES BY THE MISSISSIPPI VALLEY DENTAL ASSOCIATION.

CINCINNATI, June 12, 1860.

At a special meeting of the "Mississippi Valley Association of Dental Surgeons," held at the office of Dr. Richardson on the evening of June 12, 1860, Dr. Sam'l Wardle was called to the Chair, and Dr. T. F. Davenport elected Secretary *pro tem.*

On motion of Dr. Richardson, the Association proceeded to the special business before it, viz: the election of delegates to the "National Association" at Washington. The following gentlemen were unanimously elected:

Dr. W. H. Atkinson, Cleveland, O.; Dr. E. Collins, Connersville, Ia.; Dr. J. P. Ulrey, Rising Sun, Ia.; C. N. Woodward, Ripley, O.; J. B. Lindsay, Maysville, Ky.; H. McCullum, Augusta, Ky.

On motion, the Association adjourned.

T. F. DAVENPORT, Sec'y pro tem.

